# Addressing Global Disparities in the Burden of Noncommunicable Diseases: Call for Papers

**DOI:** 10.1371/journal.pmed.1001360

**Published:** 2012-12-27

**Authors:** The PLOS Medicine Editors

## Abstract

The PLOS Medicine editors issue a call for papers on addressing global disparities in non-communicable diseases–CVD, cancer, COPD, asthma, diabetes, mental health disorders, and substance abuse–for 2013.

Noncommunicable diseases (NCDs), most often defined as chronic medical diseases including cardiovascular diseases (heart disease and stroke), cancer, chronic respiratory diseases (chronic obstructive pulmonary disease and asthma), and diabetes [1], are responsible for two-thirds of the world's deaths, one-fourth of which occur before the age of 60 years [2]. Nearly 80% of NCD deaths occur in low- and middle-income countries (LMICs), where they are also increasing most rapidly [2]. Trends in modifiable risk factors of tobacco use, unhealthy diet, physical inactivity, and harmful use of alcohol [3] suggest these statistics will only get worse: of the estimated 1.2 billion smokers in the world, most are from LMICs, where tobacco use is rising by 3% per year [4]. People living in LMICs increased their consumption of meat, eggs, and milk by 50% on average from 1973 to 1996 [5], and more recent data show that populations in China have increased meat consumption through 2010 [6]. Sales of refined foods—including soft drinks and processed foods that are high in salt, fat, and sugar—are increasing fastest in LMICs [7], and increased income tends to shift food importation to sugar and other sweeteners [8]. While increased intake of protein and other nutrients are important for underweight individuals, saturated fats, sugars, and refined foods contribute to an unhealthy diet. The double burden of disease, in which significant numbers of overweight and underweight individuals exist within the same country [9], and often in the same household [10], is a particularly daunting challenge for LMICs.

In response to this growing burden, the United Nations held a High-Level Meeting on NCDs in September 2011 [11] and the World Health Organization (WHO) just released a Draft Global Monitoring Framework for NCDs that included global targets for the prevention and control of NCDs [3]. However, recognition has not yet translated to sufficient resources to address the burden: the WHO budget devoted to NCDs has been reduced by 20% since 2010 and comprises only 5%–8% of the total WHO budget [12]. Less than 3% of global health aid is earmarked for NCDs, and the Bill & Melinda Gates Foundation offers less than 3% of its funding for research into NCDs [13]. Consider this relatively meager allocation next to the prospect of rapid growth of international food and tobacco markets in LMICs [13]. For example, the entire WHO budget is only half of the Coca-Cola Company's marketing budget [12]. In fact, in addition to exploiting new markets, corporations are getting a place at the table: even as Mexico has developed the highest rate of obesity and overweight and the highest consumption of Coca-Cola in the world [12], the Pan American Health Organization, a regional office of WHO, accepts money from the Coca-Cola Company, PepsiCo, Kraft, Nestle, and Unilever [12], and some members of the WHO's Nutrition Guidance Expert Advisory Group have food industry ties, including receiving funding from Unilever and Nestle [12]. Such relationships may undermine the credibility and potentially the independence public health organizations need to be effective [14].

Funding for NCDs is a different story in the high-income nations. Estimated 2012 National Institutes of Health funding for cancer and cardiovascular disease alone is more than $7 billion [15]. However, results of research conducted in the United States may not always translate to US clinical settings [16], much less into improved health in LMICs. Even in infectious diseases, where more research specific to LMICs has been conducted, studies on implementing interventions may be lacking [17]. Nevertheless, the relatively successful infectious diseases programs like HIV/AIDS treatment and prevention provide useful lessons for addressing NCDs, including improving advocacy and recognition of disease burden, national planning and resource allocation, long-term donor investment, and strengthening health systems [18].

With these global needs and disparities in mind, the editors of *PLOS Medicine* are issuing a call for papers into research and commentary on NCDs directed toward improving population health and reducing health disparities. For this theme, we include as part of the definition of NCD research the identification, treatment, and prevention of mental health and substance use disorders, because mental health disorders and substance abuse also cause considerable morbidity and mortality, contribute to NCDs, and similarly require the health systems necessary to care for chronic conditions [19]. Topics *PLOS Medicine* will consider for the theme include the outcomes, exposures, and health system responses listed in the WHO's Draft Global Monitoring Framework for NCDs [3], as well as other research in prevention, identification, and treatment of this broader definition of NCDs. We will consider studies addressing the translation of research to implementation in practice in a variety of settings, but particularly for populations with poorer health. Translation of research need not be one way; innovative solutions for addressing NCDs in LMICs may have valuable lessons for high-income countries and vice versa. We encourage authors to explore the barriers and pitfalls to effective implementation and the unique challenges of implementing specific interventions in groups with disparities.

Decisions regarding publication of research articles will be based on importance, novelty, and rigor of methodology and analysis. Commentary articles submitted for the *PLOS Medicine* Magazine must be novel and well-argued. Authors are encouraged to refer to the Author Guidelines, available at http://www.plosmedicine.org/static/guidelines.action. As with all submissions to *PLOS Medicine*, authors are asked to submit a presubmission inquiry. There is no guarantee of acceptance; high-quality research that is in scope and methodologically appropriate for consideration will undergo our usual review process. Manuscripts that are considered potentially appropriate after evaluation of the full paper will undergo review by the editors, followed by review by an academic editor, and possibly external peer review including a statistical reviewer if the manuscript passes each previous phase of review. We will publish research and commentary on NCDs throughout 2013 and beyond, as well as on other important topics of global health relevance [20].

We encourage authors to submit their best work in NCDs to help address this critically important global health challenge. Publishing this essential research and commentary via open access in *PLOS Medicine* will ensure that it is widely distributed and accessed, reaching the individuals and countries who need it the most.

## Abbreviations

LMIC: low- and middle-income country
NCD: noncommunicable disease
WHO: World Health Organization
